# Collision of Trapped Topoisomerase 2 with Transcription and Replication: Generation and Repair of DNA Double-Strand Breaks with 5′ Adducts

**DOI:** 10.3390/genes7070032

**Published:** 2016-07-01

**Authors:** Hong Yan, Margaret Tammaro, Shuren Liao

**Affiliations:** Fox Chase Cancer Center, 333 Cottman Avenue, Philadelphia, PA 19111, USA; Margaret.Tammaro@fccc.edu (M.T.); Shuren.Liao@fccc.edu (S.L.)

**Keywords:** topoisomerase 2, DNA double-strand breaks (DSBs), DSB repair, 5′ strand resection, 5′ adducts, Mre11, CtIP, DNA2

## Abstract

Topoisomerase 2 (Top2) is an essential enzyme responsible for manipulating DNA topology during replication, transcription, chromosome organization and chromosome segregation. It acts by nicking both strands of DNA and then passes another DNA molecule through the break. The 5′ end of each nick is covalently linked to the tyrosine in the active center of each of the two subunits of Top2 (Top2cc). In this configuration, the two sides of the nicked DNA are held together by the strong protein-protein interactions between the two subunits of Top2, allowing the nicks to be faithfully resealed in situ. Top2ccs are normally transient, but can be trapped by cancer drugs, such as etoposide, and subsequently processed into DSBs in cells. If not properly repaired, these DSBs would lead to genome instability and cell death. Here, I review the current understanding of the mechanisms by which DSBs are induced by etoposide, the unique features of such DSBs and how they are repaired. Implications for the improvement of cancer therapy will be discussed.

The double-helical structure of DNA dictates that topological strains are generated by replication forks and transcription machineries as they plow between the two intertwined strands [1]. An essential protein for managing DNA topology is topoisomerase 2 (Top2) [2,3,4]. Top2 is a homodimeric enzyme that changes topology by nicking the two strands of DNA to create a double-strand break (DSB), through which another DNA molecule then passes. In theory, physical breakage of DNA poses a serious danger to genome integrity and cell survival. Top2 solves this problem by forming a covalent complex between the 5′ end of the broken DNA and the tyrosine in the catalytic center (Top2cc). The strong interaction between the two subunits ensures that the 5′ ends are juxtaposed to the 3′ ends and can be quickly and faithfully resealed in situ.

Top2ccs are normally transient, but can be trapped by various factors, such as DNA lesions, natural products in the diet, chemicals in the environment and, most importantly, many anticancer drugs [2]. Prolonged trapping allows cellular processes to convert Top2ccs into true DSBs, which, if not repaired or improperly repaired, would lead to genome instability or cell death [5]. Trapped Top2ccs are an intrinsic aspect of genome maintenance, and their significance is further amplified by the central role they play in mediating the cytotoxicity of some of the most widely-used cancer drugs. This review will focus on how trapped Top2ccs are converted into DSBs, the unique features of the resulting DSBs and what pathways are employed to repair them. Emphasis will be on higher eukaryotes, and implications for cancer therapy will be explored.

## 1. How Trapped Top2ccs Are Converted to DSBs: Collision with Transcription

**Figure 1:**
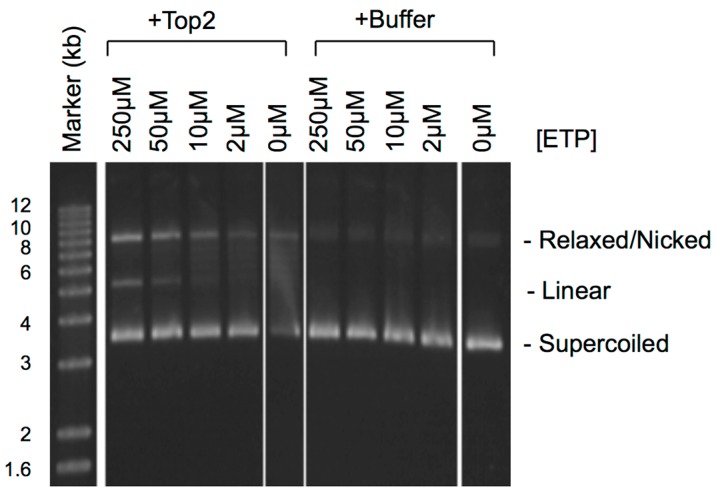
The effect of etoposide concentration on Top2cc trapping. The reactions (5 μL) contain: pGT2 plasmid DNA (10 ng/μL), Top2 (1 unit/μL; USB, OH) or buffer and the indicated concentrations of etoposide (ETP) (Sigma, St. Louis, MO, USA) (the Top2 reactions also contained 1 mM ATP). After incubation at 37 °C for 10 min, the reactions were terminated with 1.5 μL of 4% SDS + 50 mM EDTA and then treated with 7.5 μL proteinase K (1 μg/μL; Sigma, St. Louis, MO, USA) at room temperature for 2 h. The products were separated on a 1% TAE-agarose gel at 80 volts for 3 h and detected by SYBR Gold staining (Invitrogen, Carlsbad, CA, USA).

Etoposide (VP16) is a Top2-targeting drug widely used in the treatment of leukemia, lymphoma and solid tumors [3,6,7]. It binds directly to Top2 to inhibit the religation step of the catalytic cycle [8]. The two subunits act in a coordinated way, but are not absolutely dependent on each other [2]. Each subunit of Top2 can be bound by one etoposide molecule and independently inhibited [9]. Etoposide-trapped Top2ccs are thus expected to be a mixture of single-strand nicks and double-stranded DNA breaks. As shown in Figure 1, the higher the concentration of etoposide, the more Top2 is trapped in the double-nicked state, resulting in more plasmid linearization after SDS-proteinase K treatment. Top2 is both necessary and sufficient for the cytotoxicity of etoposide [10]. Unlike other Top2-targeting drugs, such as doxorubicin, etoposide does not intercalate in DNA [3,11]. The binding of etoposide to Top2 is readily reversible, and the nicks are immediately resealed after etoposide dissociates [12]. For mechanistic studies, etoposide is an ideal tool for generating trapped Top2ccs without the non-specific effects of DNA intercalation by other drugs.

In mammalian cells, the fate of etoposide-trapped Top2ccs is strongly affected by the isoforms of Top2. While lower eukaryotes have only one Top2, mammalian cells have two Top2 isoforms, Top2α and Top2β, sharing ca. 70% sequence identity [13,14,15,16]. Top2α promotes replication, transcription, chromosome structure and chromosome segregation [17]. It is essential for cell proliferation and expressed mostly in dividing cells during the S and G2 phases [18,19,20]. In contrast, Top2β participates mainly in transcription and is expressed in both dividing and non-dividing cells [21]. It is dispensable for cell proliferation, but required for neural development in mice [22,23,24]. The two isoforms use the same catalytic mechanism and are equally inhibited by etoposide [25,26]. However, their distinct biological functions and expression profiles have a profound impact on the mechanisms by which etoposide-trapped Top2α and Top2β are processed to DSBs in cells.

Trapped Top2cc is expected to block the progression of replication and transcription machineries. It has been observed that trapped Top2cc is rapidly degraded by the 26S proteasome in a transcription-dependent, but replication-independent manner [27,28,29]. The requirement for transcription is not due to new protein synthesis, suggesting that it is the collision with transcription that acts as the trigger for Top2cc degradation. ICRF-193, a catalytic inhibitor that traps Top2 on DNA after the resealing of nicks, has the same effect, suggesting that the degradation is not a response to DSBs [30]. In principle, if both subunits are inhibited, Top2cc degradation should expose the nicks on both strands of DNA, resulting in the formation of a true DSB. In support of this hypothesis, inhibiting either transcription or the 26S proteasome dramatically reduced the number of etoposide-induced DSBs based on the neutral COMET assay [31]. Both isoforms of Top2 can be degraded, but degradation of Top2β is much more rapid and extensive than that of Top2α. In agreement with this difference, DSB induction by etoposide is essentially blocked in mouse Top2β^−/−^ cells based on the neutral COMET assay [32]. Collectively, these and other similar observations have led to the model that collision between trapped Top2βccs and transcription is the mechanism by which DSBs are induced by etoposide and doxorubicin [32,33]. More recent studies have further elucidated some key mechanistic details of this model. When RNA polymerase II (RNAPII) is stalled by a Top2βcc, it recruits the 19S AAA ATPases of the 26S proteasome, which then channel the Top2βcc to the 20S proteasome for degradation in a ubiquitin-independent way [34].

## 2. How Trapped Top2ccs Are Converted to DSBs: Collision with Replication

The finding that transcription and Top2β play key roles in DSB induction is not surprising, but fully consistent with the known properties of Top2β and etoposide. What is surprising is the apparent irrelevance of Top2α and replication. Many studies have shown that Top2α, the major isoform in proliferative cells, rather than Top2β is the dominant isoform mediating cytotoxicity of etoposide [31,35,36]. Even in the study showing that Top2β is the isoform mediating DSB induction, Top2α is still the isoform responsible for the cytotoxicity of etoposide [32]. Replication, which in principle can also collide with trapped Top2ccs, has also been long known to partially mediate the cytotoxicity of etoposide [37,38]. In one study, inhibiting replication is significantly more effective than inhibiting transcription in protecting cells from etoposide [39].

The solution to this apparent paradox most likely lies in the assay used to detect DSBs. In the studies that elucidate the Top2β-transcription mechanism, DSBs are detected by the neutral COMET assay [31,32,33,34]. This assay is based on the principle that broken genomic DNA strands in cells embedded in agarose have a faster mobility than intact genomic DNA during electrophoresis under non-denaturing conditions. The sensitivity is determined by the size and structure of DNA. If there are not enough DSBs, genomic DNA would still be too large to migrate into agarose. Furthermore, genomic DNA undergoing replication usually carries bubbles or branches, structures known to dramatically slow down electrophoresis mobility [40,41]. In the Top2β-transcription studies, a very high concentration of etoposide (250 μM) was used, far more than required for cytotoxicity [32]. This ensures more Top2ccs to be inhibited at both subunits, resulting in more DSBs after transcription-stimulated degradation. Top2α is a key protein for replication, but the COMET assay might not be sensitive enough for detecting S phase DSBs, even if there is a replication-dependent mechanism. In addition, transcription-stimulated degradation of Top2α is much less efficient than that of Top2β. As such, the COMET assay is expected to detect predominantly DSBs generated by the Top2β-transcription mechanism. To detect DSBs that might be generated by Top2αccs and/or replication, a more sensitive assay unaffected by replication bubbles or branches is required.

**Figure 2:**
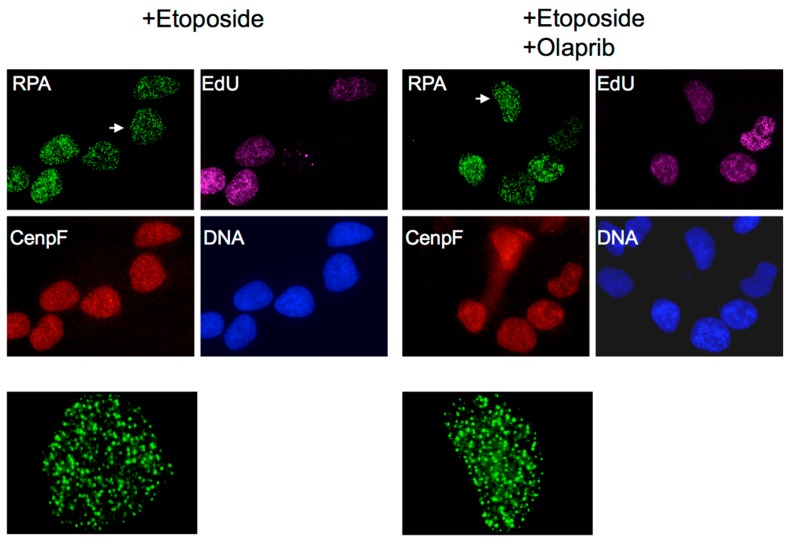
Olaparib does not affect the induction of RPA foci by etoposide. Human osteosarcoma U2OS cells treated with 250 μM etoposide for 2 h in the presence or absence of 50 μM olaparib (Selleckchem, Houston, TX, USA). Olaparib and 5-ethynyl-2′-deoxyuridine (EdU; Invitrogen; Carlsbad, CA, USA) were added 30 min and 15 min, respectively, before etoposide. Cells were fixed and stained for RPA, EdU, CenpF, and DNA as previously described [44]. CenpF expression begins in the S phase and peaks in G2.

Etoposide has long been known to rapidly induce a large number of discrete subnuclear foci containing the eukaryotic ss-DNA binding protein replication protein A (RPA) [42,43]. They are formed in the S and G2 phase, but not in G1 phase cells [44]. This cell cycle profile is reminiscent of that of DSB resection, an S- and G2-specific process that degrades the 5′ strand to form 3′ ss-DNA for homology-dependent repair (HDR) [45]. ICRF-193 does not induce such RPA foci, supporting that their formation requires the DNA first to be nicked by etoposide-induced trapping [46]. They are insensitive to olaparib, an inhibitor of poly(ADP-ribose) polymerase (PARP) (Figure 2), suggesting that the S phase RPA foci are not the result of resected reversed replication forks accumulated in the absence of PARP activity [47,48]. Finally, the RPA foci can be removed by *Escherichia coli* ExoI, a 3′- > 5′ ss-DNA exonuclease, but not by RecJ, 5′- > 3′ ss-DNA exonuclease [46]. This is consistent with the direction of DSB resection, which results in 3′ ss-DNA. Collectively, these observations suggest that etoposide-induced RPA foci represent RPA molecules bound to the 3′ ss-DNA of resected DSBs.

**Figure 3:**
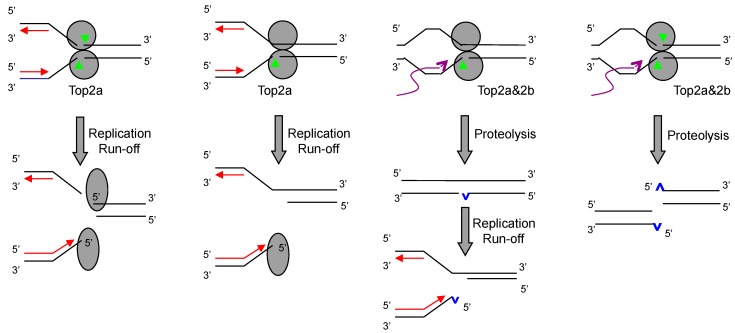
Model for the replication-dependent and transcription-dependent induction of DSBs by etoposide. Upon collision with the replication fork, ss-Top2αccs and ds-Top2αccs are converted into DSBs by replication run-off. Upon collision with the transcription machinery, Top2ccs are degraded by the 26S proteasome, resulting in DSBs and SSBs. Unrepaired SSBs can also be converted to DSBs by replication run-off.

RPA foci can therefore be used as a simple yet sensitive readout for DSB induction. Using this assay, the mechanism of DSB induction by etoposide is re-investigated [44]. It is found that etoposide induces DSBs predominantly in S phase cells at low concentrations. The induction is completely dependent on Top2α and is blocked by inhibitors of replication, but not of transcription or 26S proteasome. At high concentrations, DSBs are induced in both S and G2 phase cells. The induction is now dependent on both Top2α and Top2β. In S phase cells, RPA foci are blocked only if both replication and transcription (or 26S proteasome) are inhibited. In G2 cells, RPA foci are blocked by inhibitors of either transcription or 26S proteasome, but not of replication. These observations support a model that etoposide-induced DSBs are generated by both a replication-dependent and a transcription-dependent mechanism (Figure 3). At low concentrations of etoposide, the majority of Top2ccs are trapped in the single-strand nicks state (ss-Top2ccs) [49,50]. Transcription-stimulated degradation of ss-Top2ccs would result in single-strand breaks (SSBs) rather than DSBs. However, collision with the replication machinery would convert ss-Top2ccs into DSBs, in a way similar to that for Top1ccs [51]. At high concentrations of etoposide, more Top2ccs are trapped at both subunits to form ds-Top2ccs. Replication can convert Top2ccs into DSBs, but so can transcription-stimulated degradation. Top2α participates in DNA replication and is therefore the major mediator for the replication-dependent mechanism. Top2β participates in transcription and is a mediator for the transcription-dependent mechanism. However, Top2α can also mediate the transcription-dependent mechanism, making it overall the major isoform mediating DSB induction by etoposide. Because DSBs are far more lethal than SSBs [52], this explains why Top2α is the major isoform mediating the cytotoxicity of etoposide in proliferative cells.

## 3. Do Top2ccs-Derived DSBs Carry 5′ Adducts?

Among all types of DSBs, Top2-derived DSBs are considered unique in that they carry adducts in the form of degraded (down to a small peptide) or intact Top2 at the 5′ end. This type of DSB is also formed during meiosis by the Spo11 protein, which naturally lacks the resealing activity and becomes irreversibly cross-linked to the 5′ end [53]. While there is physical evidence for DSBs carrying 5′ Spo11 [54], the evidence for DSBs carrying 5′ Top2 is less direct. Assays such as trapped in agarose DNA immunostaining (TARDIS) and immunocomplex of enzyme (ICE) have demonstrated that etoposide traps Top2 in a covalent complex with DNA in cells [26,55]. However, these assays by themselves cannot distinguish ds-Top2ccs from ss-Top2ccs or Top2ccs prior to DSB conversion from Top2ccs post-DSB conversion. Based on the mechanism of transcription-mediated DSB induction, one can safely deduce that the resulting DSBs must carry a degraded Top2 at the 5′ end. If an ss-Topcc is degraded, it is also reasonable to deduce that the exposed nick, if not repaired, would be converted to a similar type of DSB by replication run-off (a hybrid mechanism that is both transcription dependent and replication dependent). A strong support for the existence of this type of DSB is that an enzyme, tyrosyl-DNA phosphodiesterase 2 (Tdp2), has been identified to specifically cleave off the degraded Top2 located at the 5′ end [56]. If the adduct is placed at an internal nick, the activity of Tdp2 is dramatically reduced [57]. Consistent with the enzymatic activity, cells mutated in Tdp2 are hypersensitive to etoposide [56,58,59,60].

The structure of ends produced by the collision between replication and intact Top2ccs is less certain. DSBs might be formed by simple replication run-off, resulting in Top2cc at the 5′ end; or they might be formed by nucleolytic processing of stalled forks, placing Top2cc at an internal position. The distinction between the two can be deduced from the enzymatic property of another nuclease, Mre11. Mre11 (as part of the (MRX) complex; Mre11-Rad50-NBS1 (MRN) in higher eukaryotes) can be activated by Sae2 (CtIP in higher eukaryotes) to nick the 5′ strand close to the end, but only when the end carries a bulky adduct, such as streptavidin [61]. Intact or largely intact Top2ccs trapped by etoposide can be efficiently released from chromatin by Mre11 and CtIP-dependent reactions [62,63,64]. These observations lend strong support for the existence of DSBs with an intact Top2 at the 5′ end.

## 4. What Pathways Repair DSBs with 5′ Adducts?

**Figure 4:**
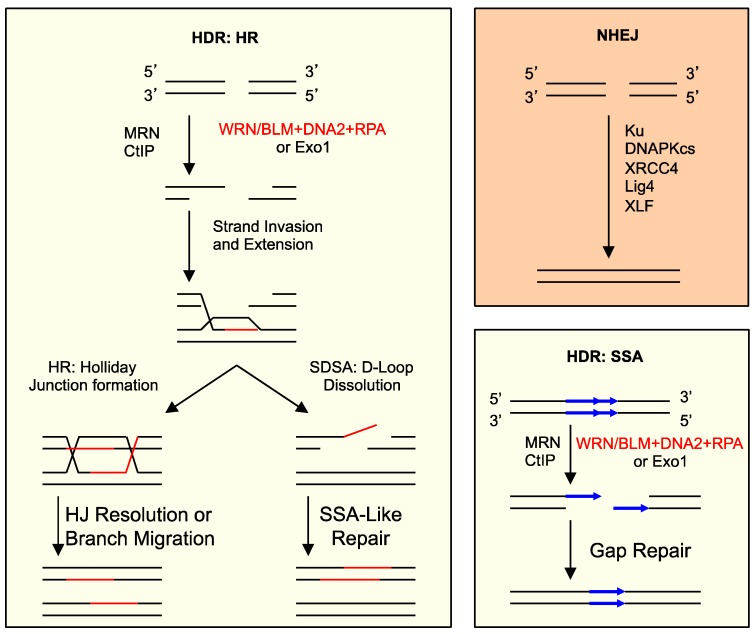
Major DSB repair pathways and 5′ strand resection in eukaryotes. Non-homologous end joining (NHEJ) involves limited or no processing of ends. Homology-dependent repair (HDR) consists of homologous recombination (HR) and single-strand annealing (SSA), both of which depend on 5′ strand resection and sequence homology for repair. Resection is carried out by either the 5′- > 3′ ds-DNA exonuclease Exo1 or the combined actions of a RecQ-type DNA helicase, the DNA2 nuclease and RPA. MRN and CtIP are required for the initiation of resection. SDSA: synthesis-dependent strand annealing; HJ: Holliday junction.

There are two general pathways for DSB repair: non-homologous end joining (NHEJ) and homology-dependent repair (HDR) (Figure 4) [65,66,67]. NHEJ is essentially re-ligating the ends regardless of sequence homology, usually after minor processing. HDR depends on sequence homology and consists of two sub-pathways. One is homologous recombination (HR), which repairs DSBs by copying the missing information from a homologous sequence. The other is single-strand annealing (SSA), which can occur between two direct repeat sequences to effectively delete one of the repeats. The key event in the choice between NHEJ and HDR is the processing of DNA ends [45]. NHEJ involves no or limited processing, but HDR requires extensive resection of the 5′ strand to form a 3′ ss-tail. The Ku70-Ku80 complex orchestrates NHEJ, whereas the Mre11/Rad50/Nbs1 complex (MRN) initiates resection. The actual degradation of the 5′ strand is carried out either by the 5′- > 3′ ds-DNA exonuclease Exo1 or by the combined actions of a RecQ-type DNA helicase, the DNA2 nuclease and the ss-DNA binding protein replication protein A (RPA) [68,69,70,71,72,73].

In higher eukaryotes, NHEJ is the major repair pathway for DSBs, and DSBs induced by Top2-targeting drugs are no exception [60,74,75]. However, adducts in the form of degraded or intact Top2 at the 5′ end do pose unique challenges to the NHEJ machinery. Top2-linked DSBs trapped by etoposide cannot bind and activate DNA-PK [76]. Tdp2 has to first remove the degraded Top2 from the 5′ end before the core NHEJ proteins can access the cleaned ends to complete repair. Accordingly, cells defective in Tdp2 activity are highly sensitive to etoposide [56,58,59,60]. In contrast, cells defective in Artemis, another nuclease involved in the processing of ends for NHEJ, display no hypersensitivity to etoposide, confirming that Artemis cannot substitute for Tdp2 in the NHEJ repair of DSBs with 5′ degraded Top2 [77,78].

**Figure 5:**
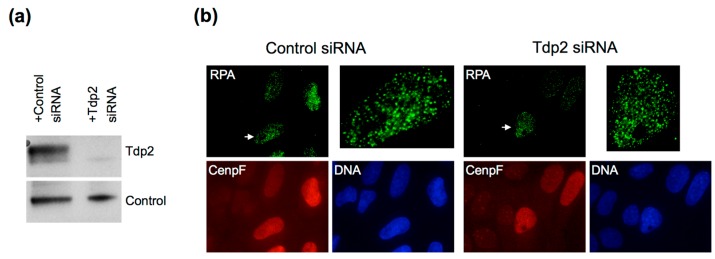
The resection of etoposide-induced DSBs does not require Tdp2. (**a**) Western blot for Tdp2 in U2OS cells treated with Tdp2 siRNAs (sc-60566; Santa Cruz Biotech, Dallas, TX, USA) or control siRNA (D-0012101-03; GE Dharmacon, Lafayette, CO, USA) following the published procedure [44]. Tdp2 was detected with rabbit polyclonal anti-Tdp2 antibodies (Bethyl Laboratories, Montgomery, TX, USA); (**b**) RPA foci induced by etoposide in U2OS cells treated with control siRNA or Tdp2 siRNAs. Cells were exposed to 250 μM etoposide for 2 h, fixed and stained for RPA, CenpF and DNA as previously described [44]. Close-ups of the nuclei indicated by the arrows are shown on the upper right of each panel.

What Tdp2 cannot remove is intact Top2 at the 5′ end, suggesting that NHEJ might not be the only pathway to repair etoposide-induced DSBs. In yeast *S. cerevisiae*, which naturally lacks Tdp2 activity and contains only a weak NHEJ pathway, HDR is critical for repairing DSBs induced by Top2-targeting drugs [79]. In human cells, there is also evidence for homology-dependent pathways in the repair of etoposide-induced DSBs [36]. In the chicken B cell line DT40 cells, knockout of Brca2, a critical player in HR, causes as much sensitivity to etoposide as Tdp2 knockout [60]. As introduced earlier, the key event in the choice between NHEJ and HDR is the initial processing of ends. The fact that etoposide induces RPA foci suggests that at least some of the induced DSBs are channeled to resection. Depletion of Tdp2 in cells does not inhibit the formation of RPA foci, suggesting that prior processing of the 5′ end by Tdp2 is not important for resection (Figure 5). Instead, Mre11 appears to be the key protein for removing Top2 from the 5′ end. The Mre11 endonuclease activity can be activated by Sae2/CtIP to nick the 5′ strand ca. 15 nucleotides inside a terminal bulky adduct [61]. However, it is unknown if this activity actually leads to extensive 5′ strand resection. Mre11 is also found to be required for the removal of intact Top2ccs from chromatin in etoposide-treated cells or reconstituted nuclei [62,63,64]. In addition, CtIP and Brca1, as well as their physical interaction are critical for Top2 removal [64,80]. Interestingly, the nuclease domain of human Mre11 is shown to be sufficient to remove Top2α adducts [59]. This suggests that the ability of Mre11 to clip off 5′ adducts is normally suppressed by the rest of the MRN complex, and CtIP’s function is to relieve this suppression. However, these studies cannot distinguish Top2ccs linked to the 5′ terminus of DSBs (post-DSB formation) and Top2ccs not yet processed into DSBs (pre-DSB formation) or Top2ccs linked to nicks.

To more rigorously study the repair of DSBs with 5′ adducts, linear DNA substrates carrying 5′ adducts mimicking degraded Top2 (with phosphotyrosine (p-Tyr)) and intact Top2 (with avidin) were prepared and incubated in *Xenopus* egg extracts [46,81]. Both substrates are efficiently resected, presumably for HDR. Resection is absolutely dependent on Mre11, but the two substrates have different requirements for the nuclease activity of Mre11 and CtIP. While the 5′ avidin DNA is dependent on the nuclease activity of Mre11, the 5′ p-Tyr DNA is not. Similarly, CtIP is essential for the resection of 5′ avidin DNA, but can be bypassed with excess MRN for the resection of 5′ p-Tyr DNA. Without the Mre11 nuclease activity or CtIP, avidin cannot be removed from the 5′ end. Extensive resection is carried out mainly by DNA2, which acts after the removal of avidin. Together, these in vitro biochemical studies demonstrate that DSBs with 5′ adducts can be efficiently channeled to resection. They also suggest that the Tdp2-mediated NHEJ is not the only way to repair DSBs with degraded Top2. In S and G2 cells, these DSBs can also be repaired by HDR.

## 5. Implications for Cancer Therapy

Like all cancer drugs, the efficacy of etoposide varies widely among different types of cancer. It is also associated with the side effect of secondary leukemia as a result of drug-induced chromosome translocations [82,83]. The understanding of how DSBs are induced by etoposide and repaired by cells has strong implications for maximizing the therapeutic efficacy of etoposide while minimizing its side effects. In current therapeutic regimens, plasma levels of etoposide range from 10 μM to 130 μM [84]. At low concentrations (i.e., 10 μM), DSB induction by etoposide is mainly mediated by Top2α using the replication-dependent mechanism and thus limited to proliferative cells. At high concentrations, DSB induction can also be mediated by Top2β using the transcription-dependent mechanism and, therefore, in both proliferating and post-mitotic cells. Since Top2α is the major isoform responsible for cytotoxicity, continuous or frequent administration of low doses of etoposide is expected to kill tumor cells, which contain high fractions of S phase cells, without inflicting significant damage on normal tissues, such as heart, which are composed mostly of non-replicating cells. Furthermore, since Top2β appears to be the major isoform mediating chromosomal translocations [32,85], lower doses should also reduce the risk of secondary malignancies.

Another implication is that the efficacy of etoposide can be increased when used in combination with inhibitors of DSB repair. Inhibitors of DNA-PK and ligase IV have been identified and demonstrated to enhance the cytotoxicity of etoposide [86,87]. Efforts to identify inhibitors of Tdp2 are being actively pursued, but so far, none have shown a synergistic effect with etoposide [88,89]. Considering that Tdp2-mediated NHEJ is also required for the repair of etoposide-induced DSBs in non-proliferating cells, inhibitors of HDR might be a better choice for synergistic use with etoposide. For example, mirin, an inhibitor of the Mre11 nuclease activity [90,91], can block the removal of Top2ccs [58]. Another example is the small molecule, 4-hydroxy-8-nitroquinoline-3-carboxylic acid, which inhibits DNA2 [92], which is required for the survival of cells treated with etoposide [46]. These inhibitors or their derivatives might be able to specifically increase etoposide’s killing of proliferating cancer cells. Future studies testing these ideas could lead to the development of new drugs that act synergistically with etoposide to more effectively treat cancer.

## 6. Conclusions

The Top2 molecules trapped on DNA by the cancer drug etoposide are converted to DSBs by two mechanisms in cells. One mechanism is dependent on the collision with replication complexes, which causes replication run-off, generating DSBs with an intact Top2 linked to the 5′ end. The other mechanism is dependent on the collision with transcription complexes, which stimulates the degradation of Top2, generating DSBs with a small Top2 peptide linked to the 5′ end. The replication-dependent mechanism is mediated by Top2α and can occur even if only one subunit is trapped. In contrast, the transcription-dependent mechanism requires both subunits to be trapped and is mediated by both Top2α and Top2β. The small Top2 peptide at the 5′ end of DNA can be cleaved off by Tdp2, and the resulting ends are repaired by NHEJ. However, intact Top2 at the 5′ end is not a substrate for Tdp2 and has to be removed by the Mre11 nuclease, leading to 5′ strand resection and HDR repair. Using low concentrations of etoposide to induce DSBs primarily by the Top2α-mediated and replication-dependent mechanism is predicted to be a better strategy to maximize the preferential killing of highly proliferative cancer cells and minimize the Top2β-mediated side effects on oncogenic chromosomal translocations and post-mitotic cell survival.
